# Understanding the role of the hippocampus in representing multimodal stimuli

**DOI:** 10.1371/journal.pbio.3004058

**Published:** 2026-11-04

**Authors:** Halle R. Dimsdale-Zucker

**Affiliations:** Department of Psychology, University of California, Riverside, Riverside, California, United States of America

## Abstract

Although experiences are multimodal, much of the work on the hippocampus has focused on visual stimuli. This Primer explores a new study in PLOS Biology that presented unimodal and audiovisual stimuli and found evidence for hippocampal involvement in representing both.

## Teaser

Although experiences are multimodal, much of the work on the hippocampus has focused on visual stimuli. A new study in *PLOS Biology* presented unimodal and audiovisual stimuli and found evidence for hippocampal involvement in representing both.

Much of what we know about how the brain represents memories comes from studies of impoverished stimuli (e.g., words or pictures presented in isolation) that do not capture the complexity of our everyday experiences [1]. A long history of work has suggested a role for the hippocampus, a key memory-related brain structure tucked inside the temporal lobes of the brain, in representing and remembering visual information such as pictures of objects and complex scenes as well as linking information (or “binding”) experiences to their surrounding contexts [2]. The ability to bind or link together experiences is one of the processes that helps to support our autobiographical memories for rich everyday experiences such as coffee with a friend. Furthermore, memory-related representations in the hippocampus have been shown to both be able to represent specific memories (such as when you had coffee with your friend Val at Mundial Coffee) as well as represent commonalities across similar events (e.g., all the different times we have hung out with your friend Val or similarities amongst all of the times you went to Mundial Coffee or even across visits to many different coffee shops). Yet, a disconnect exists between the way in which highly-controlled laboratory studies are designed that often strip down a memory to its component parts versus the rich, dynamic, multisensory experiences that compose our everyday lives.

One way cognitive neuroscientists have tried to ameliorate this is by using stimuli that more closely approximate our everyday experiences, such as television shows and movies. Although this work has helped to close the gap between controlled laboratory work using relatively impoverished stimuli presented in isolation and complex real-world experiences, we do not yet understand which features of multimodal stimuli (e.g., sights, smells, sounds, etc.) are represented by the hippocampus nor how the combination of these features changes these representations. In a new study in *PLOS Biology*, Raccah and colleagues tried to disentangle hippocampal representations for the same complex stimuli by selectively decomposing visual-only or auditory-only information from the same stimulus or presenting conjoint visual/auditory information where these sensory streams were either congruent with one another (i.e., audio and visual streams from the same stimulus) or incongruent with one another (i.e., audio and visual streams from different stimuli) [3; see Fig 1 for a task and analysis schematic].

**Fig 1:**
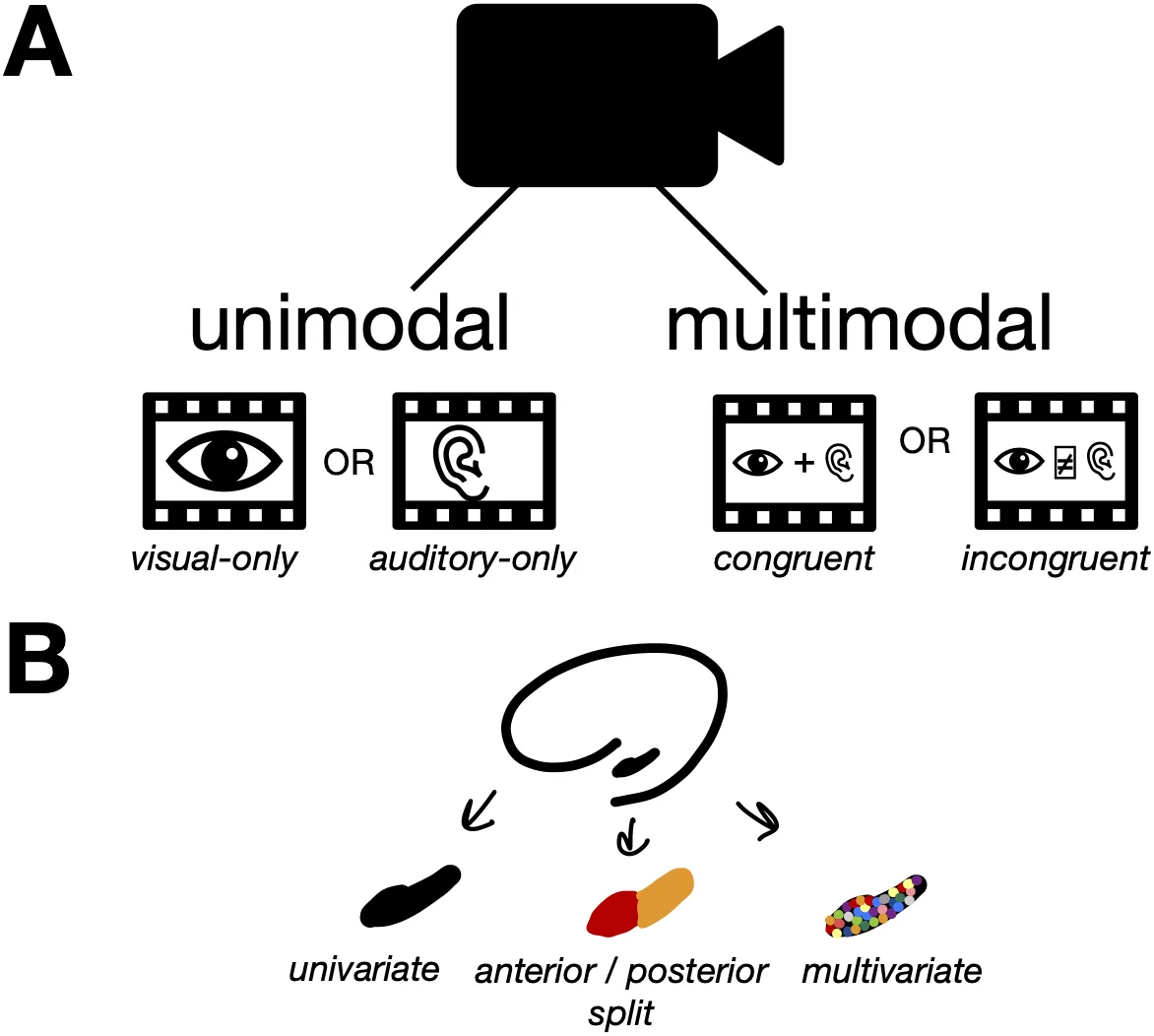
Schematic of task and overall analysis approach. **A.** While undergoing brain scanning, participants watched video clips in either unimodal (visual- or auditory-only information) or multimodal format. For multimodal clips, audio and visual components were from the same (congruent) or different (incongruent) video. **B.** Cartoon schematic of the brain (shown from a sagittal, or side, view) with the hippocampus shown. Below the brain figure, the hippocampus is drawn separately to depict the three ways the fMRI data was analyzed within this structure. (Figure drawn by HDZ).

To do this, they asked participants to repeatedly view 10 short (6 second) video clips while undergoing brain imaging (functional magnetic resonance imaging [fMRI]). These were videos of real-world experiences such as a ping-pong game or animals making noises (birds chirping, wolves howling), among others. To test their critical question of whether the hippocampus prioritized unimodal or multimodal information, they presented video clips in one of two conditions: For unimodal presentations, participants experienced either only the visual content (e.g., watching a wolf crane its neck and look up at the sky) or only the auditory content (e.g., the howling noise of the wolf). For multimodal presentations, they carefully remixed the audio/visual content across all video clips such that sometimes the audio and visual information came from the same clip and thus presented congruent information. Other times, the audio/visual information was recombined across clips resulting in incongruent pairings (e.g., watching a wolf crane its neck and look up at the sky but hearing sounds of typing on a typewriter). They interrogated representations in two different ways: by looking across activity in an entire region (univariate analyses) and by looking across distributed activity profiles (multivariate analyses) [4].

Intriguingly, they found that when considering only aggregated levels of activity (univariate analyses), the hippocampus seemed to reflect either visual-only information or combined audiovisual information but not auditory information alone. However, when they interrogated the distributed activity profiles (multivariate analyses), a more nuanced perspective emerged in which the hippocampus represented audio-only and visual-only information and their combination, with only a coherent link between the audio and visual information leading to enhancement of these representations. Furthermore, when they interrogated these representations of congruent audiovisual stimuli, they found that this truly was about representing the gestalt of the audio and visual information (that is, the whole video above and beyond simply the combination of its component parts) rather than simply their unimodal representations combined. That is, representations of the congruent audiovisual stimuli were stronger than representations for auditory-only or visual-only movies and additionally helped to explain unique variation in the measured responses of the hippocampus above and beyond what could be attributed to either auditory-only or visual-only presentations.

They additionally considered whether looking across the length of the hippocampus (its “long axis”) mattered for these distributed patterns of activity given known differences in anatomical connections and gene expression [5]. The posterior hippocampus represented the movies in a way that was specific to the visual modality, whereas the anterior hippocampus represented the movies in a way that generalized across modalities, such that the audio-only and visual-only versions of the same movie produced more similar activity profiles than unimodal presentations of different movies. Taken together, this is consistent with extant findings that suggest there is a functional gradient along the hippocampus with anterior regions representing information more abstractly and posterior regions representing information in a more detailed or specific manner [6].

Given the hippocampus’ fundamental role in supporting memory, these findings are important because they help to explain how this brain structure represents the kinds of dynamic, multimodal experiences that characterize our everyday lives. By taking different analytical approaches, they additionally help to reveal the nuanced differences of aggregating activity within a region versus trying to understand the variation within a given region across both anatomical delineations (posterior, anterior) and distributed activity profiles (multivariate analyses).

The hippocampus is a fascinating structure that has historically been appreciated largely for its role in supporting memory [7]. However, more recent characterizations have highlighted its close ties to the visual system [8] and a broad role in cognitive operations ranging from attention and language to perception and prediction, among others [9,10]. The present work further suggests a need for broadening our understanding and appreciation of how the ways in which the hippocampus represents information can help us to better understand the broad role it plays in cognition and, thus, how it can support our rich, multimodal everyday experiences.
